# Curcumin Modulates DNA Methyltransferase Functions in a Cellular Model of Diabetic Retinopathy

**DOI:** 10.1155/2018/5407482

**Published:** 2018-07-02

**Authors:** Andrea Maugeri, Maria Grazia Mazzone, Francesco Giuliano, Manlio Vinciguerra, Guido Basile, Martina Barchitta, Antonella Agodi

**Affiliations:** ^1^Department of Medical and Surgical Sciences and Advanced Technologies “GF Ingrassia”, University of Catania, Via S. Sofia 87, Catania 95123, Italy; ^2^Research and Development Department, SIFI SpA, Via Ercole Patti 36, Catania 95025, Italy; ^3^International Clinical Research Center, St. Anne's University Hospital, Brno, Czech Republic; ^4^Department of General Surgery and Medical-Surgical Specialties, University of Catania, Via Plebiscito 628, Catania 95124, Italy

## Abstract

Hyperglycaemia-induced oxidative stress appears to be involved in the aetiology of diabetic retinopathy (DR), a major public health issue, via altering DNA methylation process. We investigated the effect of hyperglycaemia on retinal DNA methyltransferase (DNMT) expression in diabetic mice, using Gene Expression Omnibus datasets. We also evaluated the effect of curcumin both on high glucose-induced reactive oxygen species (ROS) production and altered DNMT functions, in a cellular model of DR. We observed that three months of hyperglycaemia, in insulin-deficient Ins2*^Akita^* mice, decrease DNMT1 and DNMT3a expression levels. In retinal pigment epithelium (RPE) cells, we also demonstrated that high glucose-induced ROS production precedes upregulation of DNMT expression and activity, suggesting that changes in DNMT function could be mediated by oxidative stress via a potential dual effect. The early effect results in decreased DNMT activity, accompanied by the highest ROS production, while long-term oxidative stress increases DNMT activity and DNMT1 expression. Interestingly, treatment with 25 *μ*M curcumin for 6 hours restores ROS production, as well as DNMT functions, altered by the exposure of RPE to acute and chronic high glucose concentration. Our study suggests that curcumin may represent an effective antioxidant compound against DR, via restoring oxidative stress and DNMT functions, though further studies are recommended.

## 1. Introduction

The growing incidence of diabetes and longer life span in the aging population point towards an increase in patients with diabetic retinopathy (DR), a diabetes-related microvascular complication which represents a major public health issue as one of the leading causes of blindness in elderly adults [1]. Clinically, DR can be classified into nonproliferative and proliferative: the first is characterized by macular oedema, while the second one may manifest as proliferative retinal neovascularization [2]. The incidence of DR appears to be higher in patients suffering from type 1 than in those with type 2 diabetes [3]. However, regardless of the type of diabetes, both hyperglycaemia and hyperglycaemia-induced oxidative stress have been identified as the major contributing factors [4, 5]. Moreover, it has been demonstrated that DR progression continues even if normal glycaemic control is restored, suggesting that the harmful effect depends on both the duration and the severity of hyperglycaemic insult [6]. Oxidative stress has been shown to alter histone modifications and DNA methylation [7], which have been further recognized as potential epigenetic mechanisms involved in the pathophysiology of DR [8–11]. The methylation process of DNA is carried out by DNA methyltransferases (DNMTs), a family comprising 5 members, of which only DNMT1, DNMT3a, and DNMT3b are catalytically active. DNMT1 is described as the maintenance methyltransferase, while DNMT3A and DNMT3B are de novo methyltransferases [12]. In mammals, methylation almost exclusively occurs at short DNA sequences, termed CpG islands, which typically contain around 5–10 CpGs per 100 bp, and up to 80% of CpG islands is localized in noncoding regions that mainly contribute to the global methylation status [12]. There are different types of repetitive sequences scattered throughout the genome (e.g., satellite repeat, short interspersed nuclear element, and long interspersed nuclear element-1 (LINE-1)). LINE-1 sequences, accounting for ≈18% of human genome, are widely used as a surrogate marker of global DNA methylation [13–16].

The effects of curcumin, a natural phenol from the rhizome of *Curcuma longa*, have been determined in animal models and in vitro systems [17]. Recent literature reports the wound healing properties of curcumin indicating the capability to accelerate the wound healing process [18]. Particularly, the anti-inflammatory and antioxidant potentials of curcumin enhance the healing process quite effectively in diabetic rats [19]. Several lines of evidence have shown that curcumin significantly decreases lipid peroxidation, increases intracellular antioxidant amount, regulates antioxidant enzymes, and scavenges hyperglycaemia-induced ROS production [20, 21]. Particularly, treatment with curcumin reduced ROS production both in retinal pigment epithelium (RPE) cells [22] and in the retina of diabetic rats [23]. However, the relying antioxidant activity of curcumin on epigenetic mechanisms has not been completely elucidated.

The present study investigated the effect of hyperglycaemia and high glucose-induced oxidative stress on retinal DNMT activity and expression, as well as on LINE-1 methylation levels. To achieve this objective, we compared the expression levels of DNMTs in the retina of diabetic and nondiabetic mice, using the Gene Expression Omnibus (GEO) datasets. In RPE cells, we analysed the time-related effect of high glucose condition on ROS production and DNMT activity and expression. Finally, we evaluated whether the antioxidant properties of curcumin may restore high glucose-induced changes of DNMT function in RPE cells.

## 2. Materials and Methods

### 2.1. Microarray Data

Microarray datasets were retrieved and downloaded from the Gene Expression Omnibus (GEO) database (http://www.ncbi.nlm.nih.gov/geo) of the National Center for Biotechnology Information (NCBI), using the keyword “diabetic retinopathy.” The GSE12610 dataset included expression microarray profiling data derived from the whole retina of adult CD1 streptozotocin- (STZ-) induced diabetic mice (3-week and 5-week) and age-matched controls. Mice with glucose levels above 250 mg/dL were considered diabetic from the date of the last injection. To get adequate amounts of RNA, four retinas (2 animals) for each group were pooled. Total RNA was extracted and processed for being hybridized on the GPL1261 platform of Affymetrix Mouse Genome 430 2.0 Array (Affymetrix Inc., Santa Clara, CA, USA). The GSE19122 dataset compared expression microarray profiling data derived from the whole retina of eight C57BL/6J STZ-induced diabetic mice and nine insulin-deficient Ins2^Akita^ mice after 3 months of hyperglycaemia, with those derived from eight controls. Total RNA was extracted and processed for being hybridized on the GPL6885 platform of Illumina MouseRef-8 v2.0 expression Beadchip (Illumina Inc., San Diego, USA) [24]. The dataset GSE55389 included expression microarray profiling data derived from the whole retina of four 8-week-old db/db diabetic mice and four age-matched lean nondiabetic controls. Total RNA was extracted and processed for being hybridized on the GPL6246 platform of Affymetrix Mouse Gene 1.0 ST Array (Affymetrix Inc., Santa Clara, CA, USA) [25]. Due to skewed distribution, for each dataset, raw data of DNMT1, DNMT3a, and DNMT3b were extracted and signal values from the selected genes were log-transformed and normalized using the MeV free software online. The difference in log-transformed DNMT expression levels between diabetic mice and controls was compared using Student's *t*-test and reported as the absolute mean difference (MD).

### 2.2. Cell Culture

The human retinal pigment epithelial cells (ARPE-19) were purchased from the American Type Culture Collection (Manassas, VA) and maintained in Dulbecco's Modified Eagle's medium (DMEM) supplemented with 10% foetal bovine serum (FBS; Gibco BRL), 100 U/mL of penicillin and 100 lg/mL of streptomycin (Gibco BRL). Cells maintained at the following glucose conditions were used:

- Cells maintained at 5.5 mM glucose for 3 weeks (normal glucose (NG) condition)
- Cells maintained at 25 mM glucose for 3 weeks (chronic high glucose (HG) condition)
- Cells maintained at 5.5 mM glucose for 3 weeks and then transferred to 25 mM glucose medium for 24 hours (Acute HG condition)

Normal glucose condition (5.5 mM) corresponded to the fasting plasma glucose level of diabetes-free subjects, while high glucose condition (25 mM) reflected 2 h after-meal plasma glucose level in diabetic patients [26–28]. To rule out the potential effect of hyperosmotic stress, cells maintained in 25 mM mannitol medium were used as osmotic control. Cells between 6 and 10 passages were used in all experiments and incubated at 37°C and 5% CO2. The medium was changed every 48 hours. A flow-chart of in vitro experiments was reported in Figure S1.

### 2.3. Curcumin Treatment

The effect of curcumin on ROS production, DNMT activity and expression, and LINE-1 methylation was evaluated in ARPE-19 cells. In brief, cells were maintained either at 5.5 mM or at 25 mM glucose concentrations or maintained at 5.5 mM glucose and then transferred to 25 mM glucose medium. After 24 hours, cells were exposed to 25 *μ*M curcumin (Sigma Aldrich, St. Louis, MO) for 6 hours, and then processed to further analyses.

### 2.4. Determination of Cell Viability

To evaluate the effect of curcumin on cell viability, the Thiazolyl blue tetrazolium bromide (MTT) assay was performed. Cells, maintained either in normal or in high glucose conditions, were seeded at a density of 2.0 × 10^4^ cells/well in a 96-well plate. Cells were then exposed to increasing concentrations (1–50 *μ*M) of curcumin for 6 h. To determine the time-dependent effect of curcumin treatment on cell viability, cells were also exposed to 25 *μ*M curcumin for 1 to 24 hours. MTT (1.6 mg/mL) was added to the cells in each well, followed by a further incubation at 37°C for 4 h. After removing the solution, cells were resuspended in 100 *μ*L of dimethyl sulfoxide (DMSO). The optical density was read at 540 nm, and the background was subtracted at 670 nm. Cell viability (%) was reported as (OD of the treated samples/OD of the control) × 100.

### 2.5. Determination of Reactive Oxygen Species (ROS)

The intracellular ROS level was determined using the Abcam cellular ROS detection assay kit (Abcam plc, Cambridge, UK). The redox-sensitive fluoroprobe 2′,7′ –dichlorofluorescin diacetate (DCFDA) is a fluorogenic dye that measures hydroxyl, peroxyl, and other reactive oxygen species (ROS) activity within the cell. After diffusion into the cell, DCFDA is deacetylated by cellular esterases to a nonfluorescent compound, which is later oxidized by ROS into 2′,7′-dichlorofluorescein (DCF). DCF is a highly fluorescent compound which can be detected by fluorescence spectroscopy with maximum excitation and emission spectra of 495 nm and 529 nm, respectively. Briefly, ARPE-19 cells were seeded at a density of 2.0 × 10^4^ cells/well in a dark, clear bottom 96-well microplate. After removing the media, cells were rinsed with 100 *μ*L/well of 1x buffer and stained by adding 100 *μ*L/well of DCFDA solution. Cells were incubated with DCFDA solution for 45 minutes at 37°C in the dark. After removing DCFDA solution, 100 *μ*L/well of 1x buffer was added and fluorescence was immediately measured (Ex/Em = 485/535 nm).

### 2.6. Nuclear Protein Extraction

Nuclear proteins were extracted from ARPE-19 cells using the Nuclear Extraction Kit (Abcam plc, Cambridge, UK). In brief, cells were grown to 70–80% confluency and removed by trypsinization following standard protocols. Cell pellets (2 × 10^6^ cells) were resuspended in 200 *μ*L of preextraction buffer and incubated on ice for 10 minutes. After centrifugation, nuclear pellet was resuspended in 400 *μ*L of extraction buffer and incubated on ice for 15 minutes, with vortexing every 3 minutes. Finally, the suspension was centrifuged for 10 minutes at 14,000 rpm at 4°C; the supernatant was transferred into a new microcentrifuge vial to measure the protein concentration of the nuclear extract. Nuclear protein quantification was performed by the Qubit fluorometer (Invitrogen) using the Qubit Protein Assay Kit.

### 2.7. DNMT Activity Quantification

Quantification of DNMT activity was performed using the colorimetric DNMT Activity Quantification Kit (Abcam plc, Cambridge, UK), suitable for measuring total DNMT activity according to the manufacturer's instructions. In brief, 7.5 ng of nuclear extracts was diluted in 50 *μ*L/well of reaction solution. The 96-well plate, including blank and positive control, was covered and incubated at 37°C for 120 min. After removing the reaction solution, each well was washed with 150 *μ*L of wash buffer for three times, and 50 *μ*L/well of the diluted capture antibody was added. The plate was covered with an aluminium foil and incubated at room temperature for 60 min. After removing the capture antibody, each well was rinsed with 150 *μ*L of the wash buffer for three times, and 50 *μ*L/well of the diluted detection antibody was added. The plate was covered with an aluminium foil and incubated at room temperature for 30 min. After the detection antibody was removed, each well was rinsed with 150 *μ*L of the wash buffer for four times, and 50 *μ*L/well of the enhancer solution was added. The plate was covered with an aluminium foil and incubated at room temperature for 30 min. After removing the enhancer solution, each well was rinsed with 150 *μ*L of the wash buffer for five times, and 100 *μ*L/well of the developer solution was added. The plate was covered with an aluminium foil and incubated at room temperature for 10 min, away from direct light. When the positive control turned to medium blue, 100 *μ*L/well of stop solution was added to stop enzyme reaction. Absorbance was read on a microplate reader within 2 to 10 min at 450 nm with an optional reference wavelength of 655 nm. DNMT activity was reported as the percentage of control.

### 2.8. Quantitative Real-Time Polymerase Chain Reaction (qPCR)

Total cellular RNA was extracted using Trizol® Reagent (Invitrogen, Carlsbad, CA, USA), and RNA was reverse transcribed to single-stranded cDNA using the SuperScript III Reverse Transcriptase (Applied Biosystems, Foster City, CA, USA) according to the manufacturer's protocols. mRNA levels were determined by qPCR with TaqMan Gene Expression Assays (Life Technologies, Monza, MB) using the 7300 Real-Time PCR System (Applied Biosystems, Foster City, CA, USA). Specific primers were used to detect DNMT1 (assay number Hs00945875_m1), DNMT3a (Hs01027162_m1), and DNMT3b (Hs00171876_m1). Threshold cycle values in each sample were used to calculate the number of cell equivalents in the test samples. The data were normalized to the values for GAPDH expression (Hs02758991_g1).

### 2.9. LINE-1 Methylation Analysis

DNA was extracted using the DNeasy Blood and Tissue kit and quantified using the Qubit dsDNA HS (High Sensitivity) Assay Kit according to the manufacturer's protocols. The methylation analysis of the LINE-1 promoter (GeneBank accession number X58075) was investigated by pyrosequencing-based methylation analysis, using the PyroMark Q24 instrument (Qiagen), after DNA bisulfite conversion. Bisulfite treatment of 20 *μ*g of DNA extracted from each sample was completed using the Epitect Bisulfite kit (Qiagen), and the converted DNA was eluted with 20 *μ*L elution buffer. The bisulfite-modified DNA was stored at −80°C until used.

A reaction volume of 25 mL was amplified by polymerase chain reaction (PCR), using the PyroMark PCR Kit (Qiagen). According to the manufacturer's instructions, each reaction mixture contained 1.5 *μ*L of bisulfite-converted DNA, 12.5 *μ*L of PyroMark PCR Master Mix 2X, containing HotStart Taq DNA Polymerase, 2.5 *μ*L of CoralLoad Concentrate 10X, and 2 *μ*L of the forward primer (5′-TTTTGAGTTAGGTGTGGGATATA-3′) and the reverse-biotinylated primer (5′-biotin-AAAATCAAAAAATTCCCTTTC-3′) (0.2 *μ*M for each) [29, 30]. HotStart PCR cycling conditions were 1 cycle at 95°C for 15 min; 40 cycles at 94°C for 30 s, 50°C for 30 s, and 72°C for 30 s; and a final extension at 72°C for 10 min. Electrophoresis of the PCR products was performed on a 2% Seakem Agarose (Lonza, ME, USA). Gels were stained with GelRed (Biotium Inc., Hayward, CA, USA) in order to visualize the amplified DNA fragment of 290 bps.

The biotinylated PCR product was purified and made single stranded to act as a template using the Pyrosequencing Vacuum Prep Tool (Biotage Inc., Charlottesville, VA, USA). The biotinylated single-stranded product was annealed to the pyrosequencing primer (5′ AGTTAGGTGTGGGATATAGT-3′) and then subjected to sequencing using an automatically generated nucleotide dispensation order for sequences to be analysed corresponding to each reaction. The pyrograms were analysed using allele quantification mode to determine the proportion of cytosine/thymine and, hence, methylated and unmethylated cytosines at the targeted position(s). The degree of methylation was evaluated at three specific cytosine followed by guanine (CpG) methylation sites, as well as the average percent methylation of the three CpG sites.

### 2.10. Statistical Analysis

All experiments were performed in triplicate for three times. Results were reported as the MD or the fold change of control. Differences were assessed by one-way-repeated measure analysis of variance (ANOVA), followed by the Bonferroni post hoc test for multiple comparisons or by Student's *t*-test for comparison of two groups. All the analyses were conducted using GraphPad version 6.0 with a significance level of 0.05.

## 3. Results

### 3.1. Analysis of DNMT Expression Using GEO Datasets

**Figure 1:**
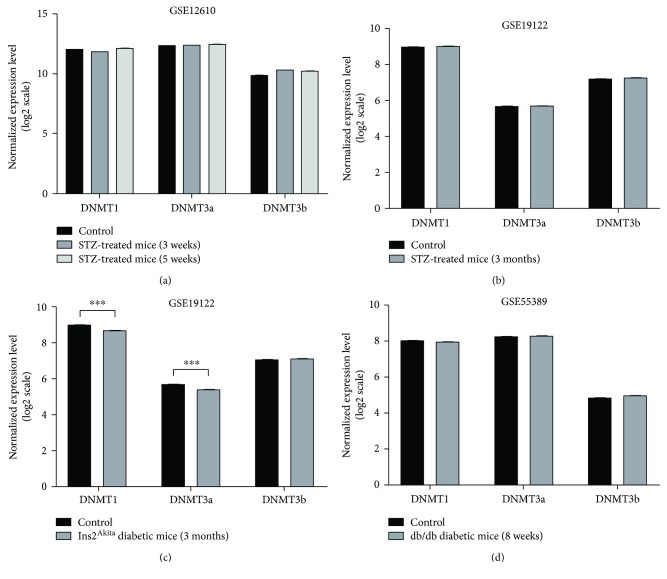
Comparison of DNMT expression using GEO datasets of microarray profiling in mouse models of diabetic retinopathy. (a) Comparison of retinal DNMT expression between adult CD1 streptozotocin- (STZ-) induced diabetic mice (3-week and 5-week) and age-matched controls (4 retinas for each group were pooled) using the GSE12610 dataset. (b) Comparison of retinal DNMT expression between eight C57BL/6J STZ-induced diabetic mice, after 3 months of hyperglycaemia, and eight controls, using the GSE19122 dataset. (c) Comparison of retinal DNMT expression between nine insulin-deficient Ins2^Akita^ mice, after 3 months of hyperglycaemia, and eight controls, using the GSE19122 dataset. (d) Comparison of retinal DNMT expression between four 8-week-old db/db diabetic mice and four age-matched lean nondiabetic controls, using the GSE55389 dataset. ^∗∗∗^ *p* < 0.001.

Differences in the expression levels of DNMT1, DNMT3a, and DNMT3b, between diabetic mice and controls at different time points, were evaluated in three distinct GEO datasets. Since DR incidence is higher in type 1 diabetes patients [3], we firstly analysed microarray data of type 1 diabetes mouse models. No significant difference was revealed by analysing the GSE12610 dataset, which compared adult CD1 STZ-induced diabetic mice, after 3 weeks and 5 weeks from induction, to nondiabetic mice (Figure 1(a)). The GSE19122 dataset reported microarray data of two type I diabetes mouse models, after 3 months of hyperglycaemia, and nondiabetic controls. Data analysis revealed that insulin-deficient Ins2^Akita^ mice, but not STZ-induced diabetic mice, showed lower DNMT1 (MD = −0.28, *p* < 0.001) and DNMT3a (MD = −0.31, *p* < 0.001) expression levels compared to nondiabetic controls (Figures 1(b) and 1c). We also analysed the GSE55389 dataset, which compared 8-week-old db/db type 2 diabetic mice to nondiabetic controls. However, no significant difference in the expression levels of DNMT1, DNMT3a, and DNMT3b was reported (Figure 1(d)).

### 3.2. High Glucose-Induced Oxidative Stress Precedes Upregulation of DNMT Expression/Activity in RPE Cells

**Figure 2:**
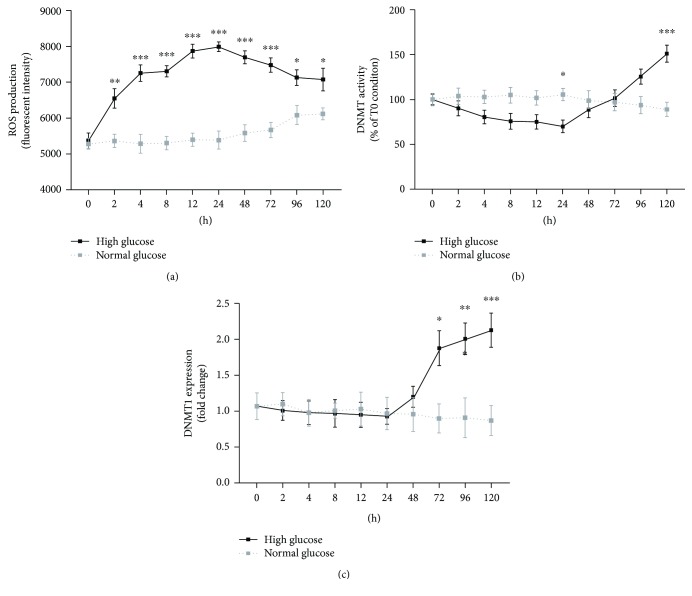
Time-dependent effects of high glucose in ARPE-19 cells. (a) Time-course analysis of ROS production in ARPE-19 cells maintained under normal and high glucose conditions. (b) Time-course analysis of total DNMT activity in ARPE-19 cells maintained under normal and high glucose conditions. (c) Time-course analysis of DNMT1 expression in ARPE-19 cells maintained under normal and high glucose conditions. ^∗^ *p* < 0.05; ^∗∗^ *p* < 0.01; ^∗∗∗^ *p* < 0.001.

One of the common features of both type 1 and 2 diabetes is hyperglycaemia-induced oxidative stress in the retina [4, 5]. Hence, we evaluated the time-dependent effect of high glucose on ROS production in ARPE-19 cells, seeded in 6-well plates and maintained either in normal (5.5 mM) or in high glucose (25 mM) condition for 5 days. Under normal glucose condition, ROS production remained stable with increasing values after 96 hours, probably due to cell confluency. Under high glucose condition, ROS production immediately increased after 2 hours, maintaining stable high levels from 2 to 24 hours and then slightly decreased. At each time point, ROS production was higher in cells maintained at high glucose compared to normal glucose condition (Figure 2(a)).

Since the analysis of GEO datasets suggested a possible time-related effect of hyperglycaemia on DNMT expression levels, we performed a time-course analysis of DNMT activity and expression in ARPE-19 cells maintained either in normal or in high glucose condition for 5 days. DNMT activity remained stable under normal glucose condition, whereas it showed a negative peak at 24 hours and a positive peak at 120 hours under high glucose condition (Figure 2(b)). Particularly, significant differences between normal and high glucose conditions were revealed after 24 (MD = −35.44, *p* < 0.05) and 120 hours (MD = 61.93, *p* < 0.001). This result was partially confirmed by time-course analysis of DNMT1 expression (Figure 2(c)). After 48 hours, DNMT1 expression level increased in cells under high glucose condition (FC = 1.25 at 72 hours, FC = 2.21 at 96 hours, and FC = 2.33 at 120 hours) but not in those under normal glucose condition. No significant difference and time-related effect were reported for DNMT3a and DNMT3b expression levels (data not shown). Overall, results from time-course analysis demonstrated that high glucose-induced oxidative stress precedes the upregulation of DNMT expression and activity, suggesting that high glucose-induced changes in DNMT function could be mediated by oxidative stress.

### 3.3. Effect of Curcumin on Viability and ROS Production in RPE Cells

**Figure 3:**
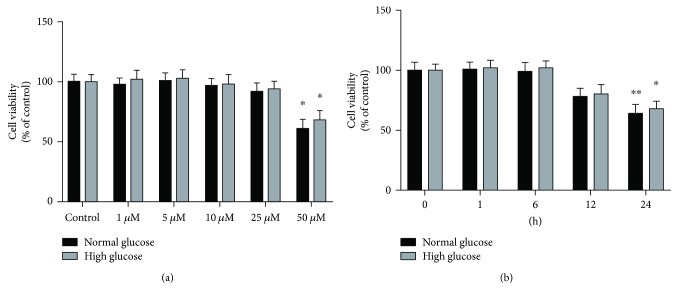
Effect of curcumin on viability of ARPE-19 cells. (a) Thiazolyl blue tetrazolium bromide (MTT) assay performed on ARPE-19 cells, maintained either in normal or in high glucose conditions, and then exposed to increasing concentrations (1–50 *μ*M) of curcumin for 6 h. (b) MTT assay performed on ARPE-19 cells, maintained either in normal or in high glucose conditions, and then exposed to 25 *μ*M curcumin for 1 to 24 hours. ^∗^ *p* < 0.05; ^∗∗^ *p* < 0.01.

**Figure 4:**
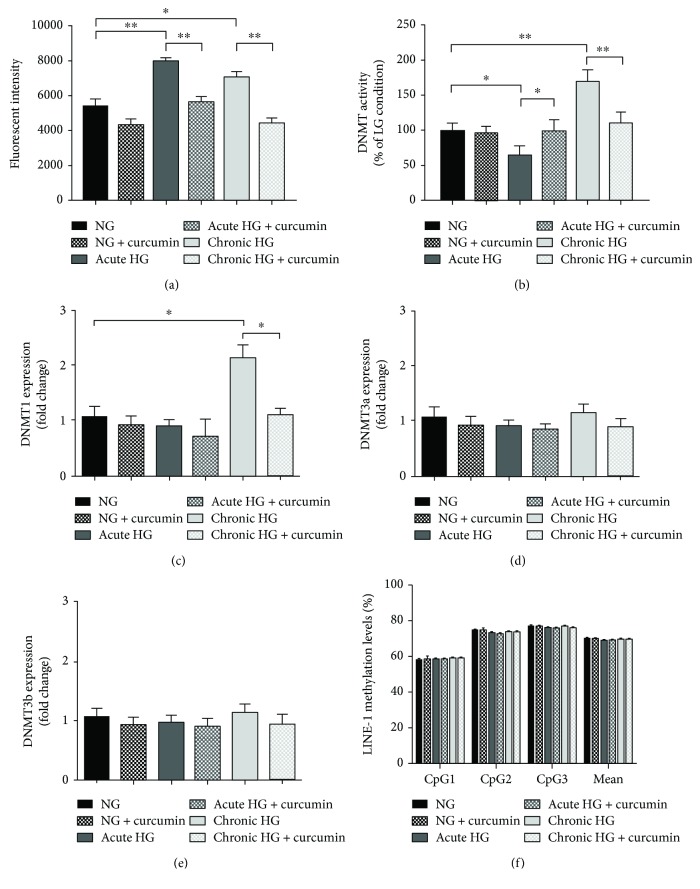
Effects of curcumin on ROS production and DNMT function in ARPE-19 cells. (a) Comparison of ROS production in ARPE-19 cells, maintained either in normal or in high glucose conditions (acute and chronic exposure), and then exposed to 25 *μ*M curcumin for 6 hours. (b) Comparison of total DNMT activity in ARPE-19 cells, maintained either in normal or in high glucose conditions (acute and chronic exposure), and then exposed to 25 *μ*M curcumin for 6 hours. (c–e) Comparison of DNMT expression in ARPE-19 cells, maintained either in normal or in high glucose conditions (acute and chronic exposure), and then exposed to 25 *μ*M curcumin for 6 hours. (f) Comparison of LINE-1 methylation level in ARPE-19 cells, maintained either in normal or in high glucose conditions (acute and chronic exposure), and then exposed to 25 *μ*M curcumin for 6 hours. ^∗^ *p* < 0.05; ^∗∗^ *p* < 0.01.

Consistently with previous studies [20–22], we aimed to evaluate the antioxidant effect of curcumin on high glucose-induced oxidative stress in RPE cells. Firstly, we determined cytotoxicity of curcumin in ARPE-19 cells, grown in a 96-well plate and then exposed to various concentrations of curcumin (1–50 *μ*M) for 6 hours or to 25 *μ*M curcumin for various durations of exposure. No significant cytotoxic effect was observed with 1–25 *μ*M curcumin, while treatment with 50 *μ*M curcumin for 6 h resulted in 39% decrease in cell viability (*p* < 0.01) (Figure 3(a)). Moreover, treatment with 25 *μ*M curcumin for up to 12 hours had no significant effect on cell viability. However, cell viability was reduced by 22% (*p* = 0.179) and 36% (*p* < 0.01) of the untreated controls after 25 *μ*M exposure for 12 and 24 h, respectively (Figure 3(b)). To avoid potential cytotoxicity, treatment with 25 *μ*M curcumin for 6 hours was chosen for further experiments. The effect of curcumin on ROS production was evaluated in ARPE-19 cells maintained at normal glucose or exposed to acute and chronic high glucose condition. Similar to time-course analysis, exposure to acute and chronic high glucose condition increased the intracellular ROS levels compared to normal glucose (*p* < 0.05 and *p* < 0.01, resp.). However, ROS production was restored by treatment with 25 *μ*M curcumin for 6 hours in both cells under acute and chronic high glucose condition (Figure 4(a)).

### 3.4. Curcumin Restores Basal Levels of DNMT Activity and Expression in RPE Cells upon Hyperglycaemic Conditions

We also evaluated the effect of curcumin on DNMT activity and expression. Compared to cells at normal glucose concentration, we confirmed a 35% decrease in DNMT activity under acute high glucose condition (*p* < 0.05); in contrast, chronic high glucose exposure led to 70% increase in DNMT activity. However, DNMT activity was restored by treatment with 25 *μ*M curcumin for 6 hours in both conditions (Figure 4(b)). With regard to DNMT1 expression level, chronic high glucose exposure up-regulated mRNA expression levels compared to cells at normal glucose concentration (FC = 2.01; *p* < 0.05). However, consistent with results on DNMT activity, treatment with 25 *μ*M curcumin for 6 hours restored DNMT1 expression level (Figure 4(c)). No significant effect of high glucose and curcumin was reported for DNMT3a and DNMT3b expression levels (Figures 4(d) and 4(e)).

### 3.5. LINE-1 Methylation Analysis

The effect of high glucose exposure and/or curcumin treatment on LINE-1 methylation, a surrogate marker of global DNA methylation, was evaluated using the bisulfite-converted DNA. Consistent with the higher expression level of the maintenance DNMT1, exposure of ARPE-19 cells to acute or chronic high glucose condition did not affect LINE-1 methylation levels. Similarly, no significant effect of treatment with 25 *μ*M curcumin for 6 hours on LINE-1 methylation levels was reported (Figure 4(f)).

## 4. Discussion

Emerging evidence suggests that pathogenesis of diabetes-related microvascular complications relies on a complex gene-environment interaction [31]. Epigenetic changes, such as DNA methylation, histone modifications, and miRNA regulation, contribute to the dysregulation of signalling pathways (i.e., oxidative stress, inflammation, apoptosis, and aging), modulating the expression of several key genes in diabetes mellitus [32, 33]. The elucidation of epigenetic changes involved in microvascular complications could improve our knowledge of pathophysiology and therapeutic management of these diseases, an important public health issue. The role of DNA methylation in vascular complications of diabetes has been recently reviewed [34]. Several lines of evidence described distinct methylation patterns in diabetes-associated cardiovascular complications [35–38], suggesting that high glucose-induced oxidative stress is an important mediator [39, 40]. Moreover, *in vitro* and epidemiological studies reported that altered promoter methylation led to the dysregulation of several genes in diabetic nephropathy [41–43].

In diabetic retinopathy, differential DNA methylation of genes involved in the natural killer cell-mediated cytotoxicity pathway was described [44]. Moreover, retinal endothelial cells exposed to high glucose concentration showed increased mitochondrial DNA methylation [8] and an imbalance between methylcytosine and hydroxyl methylation of *Matrix metalloproteinase-9* gene [45], impairing mitochondrial integrity and functions. However, in spite of substantial findings suggesting that hyperglycaemia might affect DNA methylation in the retina, the limited knowledge about the effect of high glucose in RPE is needed to be explored.

In this study, we first evaluated whether differences in the retinal DNMT expression levels existed between diabetic and nondiabetic mice, using microarray data of three distinct GEO datasets. Since DR incidence is higher in patients suffering from type 1 diabetes [3], we firstly analysed microarray data of type 1 diabetes mouse models. Data analysis did not reveal dysregulation of DNMT expression levels in 3-week, 5-week, and 3-month STZ-induced diabetic mice. Similarly, inconclusive results have been recognized analysing DNMT expression level of 8-week-old db/db diabetic mice, a genetic mouse model of type 2 diabetes. By contrast, three months of hyperglycaemia in insulin-deficient Ins2^Akita^ mice resulted in the downregulation of DNMT1 and DNMT3a expression. The Ins2^Akita^ mouse, harbouring a missense mutation in the *Insulin 2* gene, is a model for type 1 diabetes [46]. However, a previous study reported that nonobese Ins2^Akita^ mice also developed type 2 diabetes phenotypes, such as peripheral and hepatic insulin resistance and cardiac remodelling, suggesting long-term intermediate complications between type 1 and type 2 diabetes [47].

Regardless of the type of diabetes, hyperglycaemia-induced oxidative stress in the retina is one of the common features of DR pathogenesis [4, 5]. When we evaluated the time-dependent effect of high glucose in ARPE-19 cells, ROS production immediately increased after 2 hours of exposure, maintaining stable high levels from 2 to 24 hours, and then slightly decreased. Particularly, at each time point, ROS production was higher in cells maintained at high glucose compared to normal glucose condition.

Previous studies suggested that high glucose-induced oxidative stress might modulate epigenetic changes involved in the pathophysiology of DR [4, 8–11, 45, 48]. This substantial evidence, together with findings from GEO dataset analysis, prompted us to determine the effect of high glucose on DNMT function, taking into account the duration of insult. In ARPE-19 cells maintained at different glucose conditions, we demonstrated the time-related effect of high glucose exposure on DNMT activity, as shown by the time-course analysis, with a negative peak after 24 hours and a positive peak after 120 hours. Consistently, the high glucose-induced effect on DNMT expression was evident after 48 hours from the insult, with the upregulation of DNMT1. By contrast, we did not observe dysregulation of DNMT3a and DNMT3b expression.

Since DNMT1 is responsible for maintenance of DNA methylation on hemimethylated DNA [12], we also evaluated the effect of high glucose exposure on global DNA methylation, using LINE-1 methylation level as a surrogate marker. Consistent with higher DNMT1 expression, we did not observe differences in LINE-1 methylation levels between cells maintained at normal glucose concentration and those exposed to acute or chronic high glucose condition. However, the potential effects on other repetitive sequences and/or on specific promoter regions cannot be completely excluded.

Our data were in line with previous studies which demonstrated that hyperglycaemia significantly increased both DNMT activity and DNMT1 expression in retinal endothelial cells [8, 45, 49]. These changes persisted even when the glucose level is normalized, indicating that DNA methylation is probably involved in the metabolic memory of DR [11, 49–51].

This study, to our knowledge, is the first to demonstrate that high glucose-induced oxidative stress precedes the upregulation of DNMT expression and activity in RPE, suggesting that changes in DNMT function could be mediated by oxidative stress via a potential dual effect. The early effect results in decreasing DNMT activity, accompanied by the highest ROS production, while long-term oxidative stress increases DNMT activity and DNMT1 expression. It is plausible that ROS production is involved in the activation of redox-sensitive enzymes, accelerating the reaction of DNA methylation via deprotonating the cytosine molecule [52]. On the other side, it has also been demonstrated that inhibition of DNMTs, using the DNMT inhibitor RG108 (RG), protected RPE from detrimental effects of oxidative stress by the modulation of antioxidant enzyme gene expression [53]. Although the temporal relationship between high glucose-induced oxidative stress and changes in DNMT function appears evident, further *in vitro* and *in vivo* studies, using antioxidants and DNMT inhibitors, are recommended to better clarify molecular pathways involved in this mechanism.

Curcumin is considered, especially for its antioxidant properties, an interesting phytochemical candidate for the treatment of hyper-inflammatory wounds such as chronic diabetic wounds. Since it has been demonstrated that topical curcumin treatment of the wounds of diabetic rats showed enhanced angiogenesis [54], it will be interesting to evaluate the efficacy of topical curcumin on human diabetic wounds [55]. Extensive researches have increase the disease set for which curcumin may be valuable, and the identification of molecular targets will help future research in the development of curcumin as an important therapeutic agent [56]. In the present study, we also investigated whether antioxidant properties of curcumin might restore the high glucose-induced changes in RPE cells. Growing body of evidence demonstrated the pleiotropic effect of curcumin on several signalling pathways, via modulating the expression and activation of cellular regulatory systems, such as NF*κ*B, AKT, growth factors, and Nrf2 transcription factor [57–64]. Consistent with previous works [65, 66], we observed that treatment with 25 *μ*M curcumin for up to 12 hours had no significant effect on cell viability. Interestingly, we demonstrated that curcumin treatment for 6 hours reduced ROS production associated with acute and chronic exposure to high glucose concentration. In turn, the normalization of intracellular ROS levels restored the DNMT activity and DNMT1 expression. These results suggest that the antioxidant properties of curcumin might exert a beneficial effect on high glucose-induced changes in DNMT function. In line with this evidence, a previous work also demonstrated that curcumin downregulated the oxidative stress-induced expression of miR-302, an inhibitor of DNMT1 [65]. However, further studies are needed to explore if curcumin modulates DNMT function via an antioxidant effect or if it reduces oxidative stress acting on DNMT inhibition.

One of the main weaknesses of our study is that it is not evident if curcumin mainly acts as an antioxidant or DNMT inhibitor. Since curcumin treatment restored both ROS production and DNMT functions, further experiments should evaluate whether the effect of curcumin depends on the oxidative and/or DNMT pathways. Moreover, inconclusive evidence from in vivo studies exists. While we did not reveal the dysregulation of DNMT expression using microarray data of short-term type 1 diabetes mouse models, three months of hyperglycaemia in insulin-deficient Ins2^Akita^ mice resulted in the downregulation of DNMT1 and DNMT3a expression. As reported, the Ins2^Akita^ mouse is a model for type 1 diabetes, which also developed type 2 diabetes phenotypes. Overall, these findings suggest the long-term intermediate effect of type 1 and type 2 diabetes on DNA methylation, but they also point out the need for additional in vivo studies. Finally, we observed that treatment with 25 *μ*M curcumin (≈9.2 *μ*g/mL) for up to 12 hours had no significant effect on cell viability, which was consistent with previous in vitro studies [65, 66]. In addition, a previous clinical trial found that daily high-dose curcumin consumption—up to 3.6 g—was not associated with toxicity and adverse outcomes [67]. However, pharmacokinetic studies of oral Curcuma extracts in rats showed poor absorption, rapid metabolism, and elimination, which in turn suggest a low oral bioavailability [68, 69]. On the other hand, it is well established that curcumin passes through the blood-brain barrier, and dietary supplementation (≈0.2% in diet) was found to be effective against retinal degeneration in an in vivo model of light-induced retinal degeneration [70, 71]. Accordingly, further studies should be encouraged to evaluate how much diet-supplemented curcumin reaches the human retina.

## 5. Conclusions

For the first time, we demonstrated that high glucose-induced ROS production precedes the upregulation of DNMT expression and activity in RPE, suggesting that changes in DNMT function could be mediated by oxidative stress. Curcumin may represent an effective antioxidant compound to restore DNMT expression and function. However, further *in vitro* and *in vivo* studies and well-designed epidemiological studies are recommended to better clarify whether curcumin mainly acts as an antioxidant or a DNMT inhibitor.

## Data Availability

The data used to support the findings of this study are available from the co-corresponding authors upon request.

## Supplementary Materials

Supplementary Materials Figure S1: flow chart of in vitro experiments.
