# Supplementary figures and images for: Curcumin Modulates DNA Methyltransferase Functions in a Cellular Model of Diabetic Retinopathy
Source: Oxid Med Cell Longev. 2018 Jul 2;2018:5407482. doi: 10.1155/2018/5407482 (PMC6051042; doi:10.1155/2018/5407482)

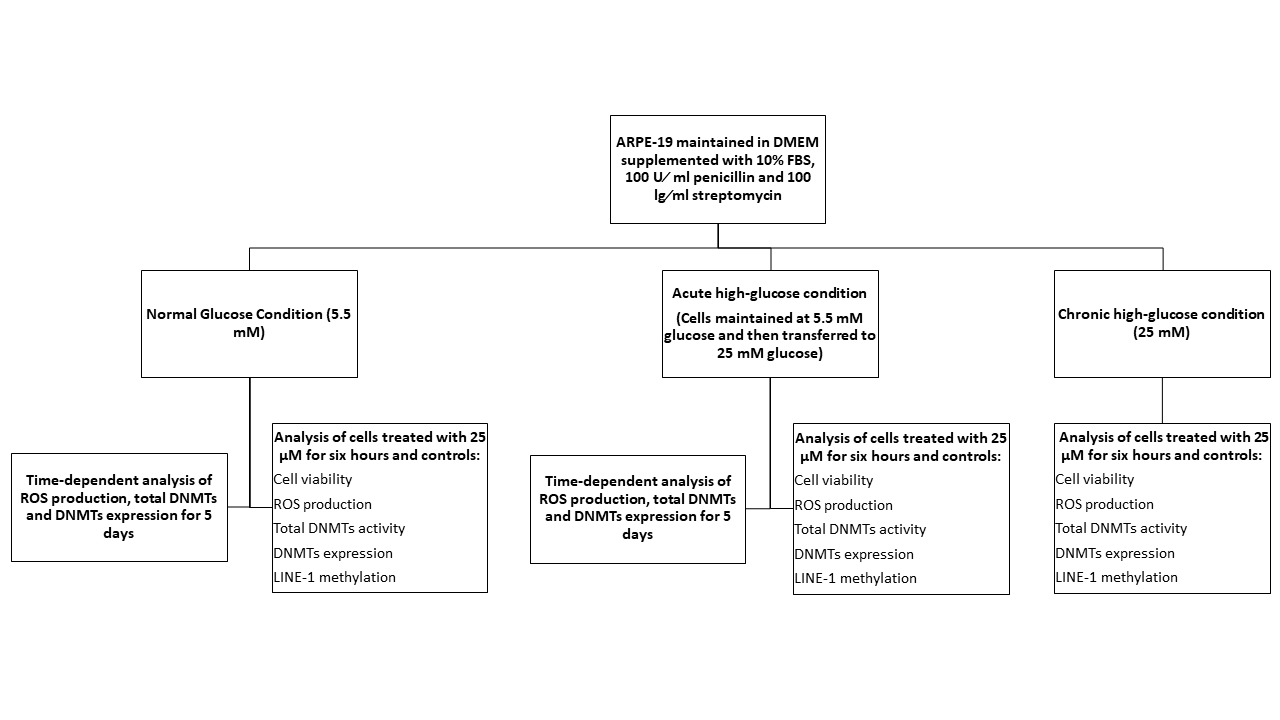

Supplement: Supplementary Materials — Figure S1: flow chart of in vitro experiments. [file 5407482.f1.tif]
